# Small cell carcinoma of the prostate after low-dose-rate brachytherapy: a case report

**DOI:** 10.1186/s13256-020-02523-5

**Published:** 2020-10-28

**Authors:** Eva Van Bos, Peter Dekuyper, Charlotte Gabriel, Marjan Waterloos, Anthony Van Baelen, Stefan Huybrechts, Filip Ameye, Antoon Lambrecht, Christof Vulsteke, Charlotte Soenens

**Affiliations:** 1 Department of Urology, AZ Maria Middelares, Buitenring-Sint-Denijs 30, 9000 Ghent, Belgium; 2 Department of Pathology, AZ Maria Middelares, Ghent, Belgium; 3 Department of Radiotherapy, AZ Groeninge, Kortrijk, Belgium; 4 Department of Oncology, AZ Maria Middelares, Ghent, Belgium; 5 Department of Molecular Imaging, Pathology, Radiotherapy and Oncology (MIPRO), Center for Oncological Research (CORE), Antwerp University, Antwerp, Belgium

**Keywords:** Prostate cancer, Small cell carcinoma, Low-dose-rate brachytherapy, Ionizing radiotherapy, Chemotherapy, Immunotherapy

## Abstract

**Background:**

Small cell carcinoma of the prostate is a rare condition with important differences from prostatic adenocarcinoma in terms of clinical and prognostic characteristics. A low prostate-specific antigen and a symptomatic patient, including paraneoplastic symptoms, characterize small cell carcinoma of the prostate. Diagnosis is made on the basis of prostate biopsy, and fluorodeoxyglucose positron emission tomography/computed tomography is often used for staging because up to 60% of patients present with *de novo* metastatic disease. Patients with metastatic disease are usually treated with platinum-based cytotoxic chemotherapy regimens similar to those used for small cell carcinoma of the lung. However, prognosis remains poor, with a median overall survival of 9 to 17 months despite therapy.

**Case presentation:**

This report describes a case of an 80-year-old Caucasian patient with lymph node and bone metastatic small cell carcinoma of the prostate following low-dose-rate brachytherapy for a low-risk prostate carcinoma and treated with chemotherapy and immunotherapy.

**Conclusion:**

Low-dose-rate brachytherapy might be an etiology of small cell prostate cancer.

## Background

Pure small cell carcinoma of the prostate (SCCP) is a rare and aggressive disorder that is distinct from the far more common prostatic adenocarcinoma [1–4]. More frequently, prostate cancer (prca) with neuroendocrine (NE) features emerges during disease progression in patients being treated or previously treated with androgen deprivation therapy (ADT) with a prevalence of 0.5–2%. However, SCCP has a prevalence of 10–20% in autopsy reports of men who died of castration-resistant prostate cancer (CRPC) [3, 4]. This means there might be underdiagnosis.

Small cell carcinoma is a distinct clinicopathologic entity that usually arises in the lung but can also originate in extrapulmonary sites, including the prostate [1, 3, 5]. SCCP has important differences from prca in terms of clinical and prognostic characteristics [1, 4]. At diagnosis, most patients are symptomatic because of the extent of the tumor. The aggressive nature and high proliferation rate lead to an increased risk for lytic or blastic bone, visceral, and brain metastases [1, 4]. Moreover, paraneoplastic syndromes such as the syndrome of inappropriate antidiuretic hormone secretion (SIADH), Cushing syndrome, and hypercalcemia are often present due to the release of peptides [1, 2, 4, 6]. Mostly, there are disproportionally low prostate-specific antigen (PSA) levels compared with the extensiveness of the disease [1, 3, 4, 6]. Pathological diagnosis is made on the basis of prostate biopsy using characteristics of small cell tumors and immunohistochemical staining for NE markers such as CD56, chromogranin A (CgA), synaptophysin, and neuron-specific enolase [1, 2]. Fluorodeoxyglucose positron emission tomography/computed tomography (FDG PET/CT) is used for staging because up to 60% of the patients present with *de novo* metastatic disease [1, 4, 7]. Given the propensity for brain metastases, magnetic resonance imaging (MRI) of the brain should be considered [1].

The optimal treatment for patients with metastatic SCCP is not established, but chemotherapy regimens containing a taxane and platinum are often used. Immunotherapy is currently used for platinum-resistant extrapulmonary small cell carcinoma (EPSCC). Up to 60% of patients have tumor reduction with receipt of carboplatin, but the duration of the response is usually short [1, 3, 6, 8, 9]. Despite chemotherapy, SCCP has a poor prognosis, with a median survival of about 10–19 months [1, 4, 6].

Case reports have been published of SCCP following high-dose-rate brachytherapy [3]. To our knowledge, this report describes the first case of SCCP following low-dose-rate brachytherapy (LDR-BT) of the prostate for a low-risk prostate tumor.

## Case presentation

In December 2011, a 73-year-old Caucasian man was admitted to our hospital with an elevated PSA of 8.59 ng/ml. His medical history consisted of cardiac stenting and pacemaker implantation. The finding of the digital rectal examination (DRE) was suspicious on the right base. His prostate volume on ultrasound was 37 ml, and his International Prostate Symptom Score was 6. Prostate biopsies showed a Gleason score of 3 + 3 = 6 prca of the right base and a Gleason score of 3 + 2 = 5 prca of the left apex. After negative staging with bone scintigraphy and CT of the abdomen, cT2aN0M0 prca was diagnosed. After a multidisciplinary discussion on the different therapeutic options, the patient decided to be treated with LDR-BT. Seventy-six seeds of ^125^I with a source activity of 0.373 mCi were implanted for a prescription dose of 145 Gy to the prostate. The first biochemical control after 4 months showed a significant drop of PSA to 0.75 ng/ml. Further oncological and clinical controls were reassuring, with a nadir PSA of 0.17 ng/ml (Table 1).

**Table 1:**
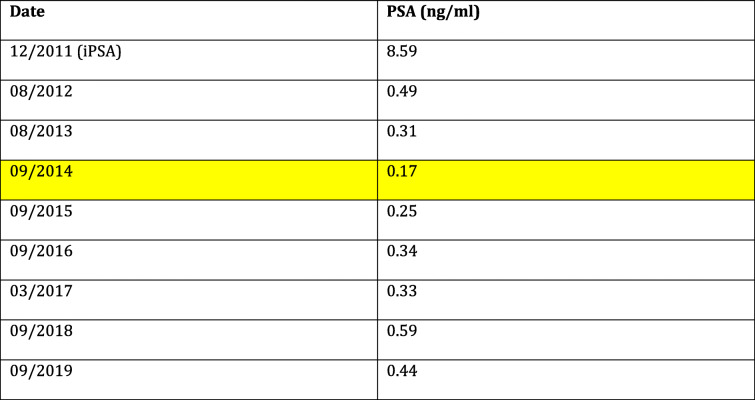
Prostate specific antigen (PSA) evolution from initial diagnosis (iPSA). The yellow bar indicates the Nadir PSA

**Fig. 1:**
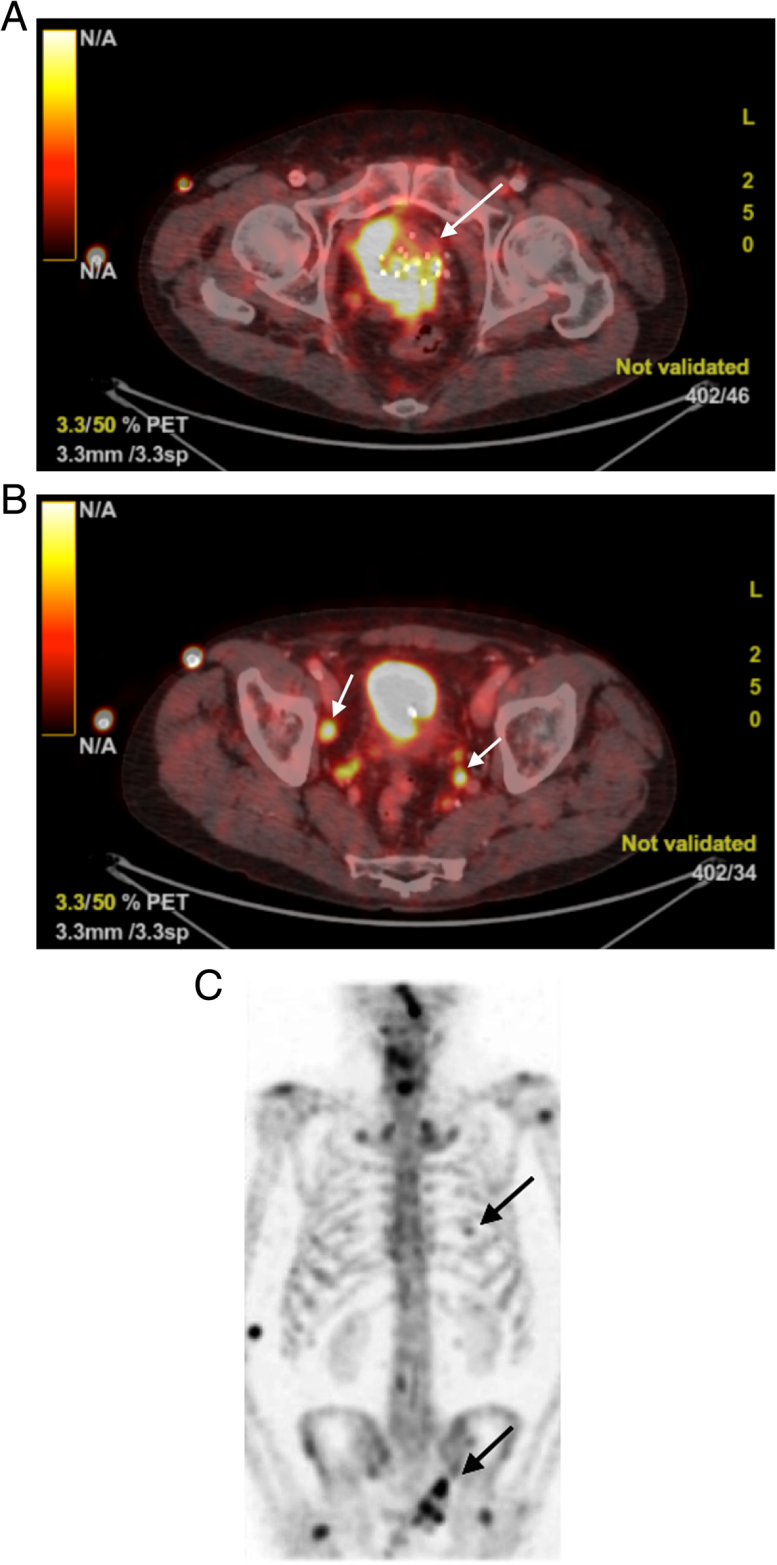
**a** Tumoral prostate with invasion of the posterior bladder wall. The arrow points to the brachytherapy seeds in the prostate. **b** Right arrow shows positive lymph nodes in the iliac region and left arrow in the obturator region. **c** Upper arrow points to osteoblastic lesions in the rib, lower arrow in the sacrum and left os ileum on bone scintigraphy

In August 2019, the patient experienced symptoms of dysuria, polyuria, and nocturia. DRE revealed an induration on the base of the prostate. The patient’s PSA concentration was 0.58 ng/ml, and the results of urinary culture and cytology were negative. The finding of cystoscopy was reassuring, except for a confined urethral stricture, which was easily dilated. MRI of the prostate could not be performed, because the patient had a non-MRI-compatible pacemaker. Prostate biopsies were scheduled. Four weeks later, a new DRE showed a manifestly increased mass. Anatomopathological examination revealed SCCP in all of the biopsies. Because pure SCCP is rare, a metastasis of SCC of the lung or prostatic invasion of SCC of the bladder was taken into account. The finding of chest CT was negative, and relook cystoscopy with random bladder biopsies under general anesthesia was planned. The cystoscopy showed no abnormalities except for tumoral bulging of the bladder trigone. The patient underwent resection of the trigone and random biopsies. The random biopsies showed eosinophilic cystitis, whereas the resected trigone was diffusely infiltrated by SCC. Histological examination of the biopsies revealed a tumor forming sheets and nests of small epithelial cells with a high nuclear/ cytoplasmic ratio, prominent necrosis, and brisk mitotic activity. Crush artefact and nuclear molding typical of small cell cancer were present in all the biopsies. Immunohistochemistry showed expression of CgA and strong expression of CD56, nuclear expression of TTF-1, and dot-like expression of CKBR. The result of PSA staining was negative, and the finding of CK20 staining was negative, excluding Merkel cell carcinoma. CT of the abdomen showed multiple adenopathies in the iliac and obturator regions. Bone scintigraphy revealed multiple osteoblastic regions at the left ninth rib, sacrum, right ischiopubic region, and left os ilium (Fig. 1). FDG-PET/CT confirmed the presence of a tumoral mass of the prostate with compression of the posterior bladder wall and positive lymph nodes in the iliac and obturator regions bilaterally (Fig. 1). The bone metastases did not capture FDG. Because of extreme lethargy and nausea, the patient had to be hospitalized and was monitored in the intensive care unit. Biochemical investigation showed severe hyponatremia of 115 mmol/L, hypo-osmolality, and high urine osmolality. The patient was diagnosed with SIADH. His symptoms and laboratory test results recovered to normal with intravenous hypertonic saline infusion and fluid restriction.

The patient’s cT4N1M1b SCCP was discussed with a multidisciplinary tumor board. Chemotherapy consisting of carboplatin (area under the curve of 5mg/ml/min, day 1) and etoposide (100 mg/m^2^ on days 1–3) plus atezolizumab (1200 mg) was started for six cycles every 3 weeks. After three cycles, a partial response was seen that was maintained after six cycles. There was a clear and rapid clinical benefit after initiation of the therapy. Nevertheless, the patient had already developed progressive disease after the second course of maintenance atezolizumab.

## Discussion and conclusion

This report presents the first case of pure SCCP following LDR-BT of the prostate for a low-risk prostate tumor. Pure SCCP is a very rare disorder in contrast to the more frequent prca with NE features (also called “aggressive-variant prca”), which emerges during the progression of prca.

Whereas the process of neuroendocrine differentiation (NED) after ADT is well known, only few cases of NED after radiotherapy have been described. Our patient developed pure SCCP almost 8 years after LDR-BT. Histological examination of the resected trigone showed all the features of small cell cancer.

NE cells are one of the three epithelial cell types in prostate tissue, accounting for < 1% [1, 10]. The physiological relevance of these cells remains controversial. The cells are androgen receptor–negative and do not proliferate themselves but induce proliferation in surrounding tumor cells by secreting peptide hormones and growth factors. Importantly, NE cells can dedifferentiate back to proliferating cells. By doing so, they contribute to tumor recurrence and proliferation [10]. Better understanding of this (de)differentiating process is needed.

Deng *et al.* studied the *in vitro* effect of ionizing radiation (IR) (40 Gy) on NED by using LNCaP prca cells (androgen receptor–positive prca cells) [10, 11]. Their main focus was cyclic AMP response–element binding protein (CREB) and activating transcription factor 2 (ATF2), which are known as activator and repressor, respectively, of NED. In 2008, they proved that IR induced NED by impairing the nuclear import of ATF2 and increasing the nuclear protein CREB [11]. In 2011, the same study group confirmed these findings in DU-145 and PC-3 cells, although to a lesser extent. To determine whether IR can induce NED *in vivo*, they conducted mouse xenograft models and used LNCaP cells that received 40 Gy of IR. Their findings were consistent with the *in vitro* results. They observed an increase in the CgA plasma level and concluded that CgA is the best biomarker for IR-induced NED because none of the nonirradiated mice had serum CgA elevation [10]. In 2014, Suarez *et al.* reported on the induction of NED by fractionated IR (40 Gy) in prca cells. They noticed two distinct phases: a radioresistant phase during the first 2 weeks, followed by a NED phase during 2 weeks. Again, increased activation of the transcription of CREB was observed. They successfully targeted CREB *in vitro* as a potential radiosensitizer and inhibitor of NED [12].

Our patient developed pure SCC in the prostate almost 8 year after initial treatment with LDR-BT. This may suggest a causal relationship between the development of SCCP and LDR-BT received at primary diagnosis, but some issues must be considered. First, the average time between diagnosis of adenocarcinoma and evolution to NE prca or SCCP reported in the literature ranges between 1 and 13 years, with a median of 20–80.4 months [13, 14]. Our patient, however, did not receive ADT in these 8 years, and in most of these studies, patients were initially treated with ADT. Second, if irradiation induces NED, one should possibly expect a much shorter duration of relapse, given the clinical behavior of SCCP. Third, the tumor might have developed independently from nonirradiated tissue. There is evidence that adenocarcinoma and SCCP have the same clonal origin. Hansel *et al.* showed a shared mutation in *TP53*, and Williamson *et al*. found a mutual rearrangement in *ERG*-*TMPRSS2* [4, 15, 16].

The optimal way to treat men with metastatic CRPC and NED is not well established. Chemotherapy regimens consisting of a taxane and platinum agent are often used. Immunotherapy has also been used for platinum-resistant EPSCC. Responses are seen in up to 60% of patients, but responses are often of short duration [1, 3, 6, 8, 9]. Likewise, our patient showed a rapid onset of clinical benefit with a partial response after three cycles, but his disease had already progressed during the second course of maintenance therapy. Nevertheless, immunotherapy approaches should be tested within clinical trials, given the recent positive results reported by Horn *et al.* showing a significantly longer overall survival and progression-free survival in extensive stage small cell lung carcinoma in the chemotherapy and immunotherapy group compared with chemotherapy alone [8].

In conclusion, to our knowledge, this is the first case report suggesting LDR-BT received in the primary setting for prca might be an etiology for the development of SCCP. Although our patient showed a short duration of response to the therapy with checkpoint inhibition, clinical trials should investigate the added value of checkpoint inhibition to chemotherapy.

## Data Availability

Data sharing is not applicable to this article, because no datasets were generated or analyzed during the current study.

## Abbreviations

ADT: Androgen deprivation therapy
ATF2: Activating transcription factor 2
CgA: Chromogranin A
CREB: Cyclic AMP response element–binding protein
CRPC: Castration-resistant prostate carcinoma
DRE: Digital rectal examination
EPSCC: Extrapulmonary small cell carcinoma
FDG PET/CT: Fluorodeoxyglucose positron emission tomography/computed tomography
IR: Ionizing radiation
LDR-BT: Low-dose-rate brachytherapy
MRI: Magnetic resonance imaging
NE: Neuroendocrine
NED: Neuroendocrine differentiation
prca: Prostate cancer
PSA: Prostate-specific antigen
SCCP: Small cell carcinoma of the prostate
SIADH: Syndrome of inappropriate antidiuretic hormone secretion
